# Seizures beget more than seizures: Understanding the cellular, structural, individual and societal impact of seizures in epilepsy

**DOI:** 10.1002/epi4.70143

**Published:** 2025-10-10

**Authors:** Matthew C. Walker, Marian Galovic, Elena Álvarez‐Barón, Adam Strzelczyk

**Affiliations:** ^1^ Department of Clinical Experimental Epilepsy, UCL Queen Square Institute of Neurology University College London London UK; ^2^ Department of Neurology, Clinical Neuroscience Center University Hospital and University of Zurich Zurich Switzerland; ^3^ Angelini Pharma Barcelona Spain; ^4^ Epilepsy Center Frankfurt Rhine‐Main, Department of Neurology Goethe‐University Frankfurt Frankfurt am Main Germany

**Keywords:** antiseizure medications, epilepsy, inflammation, quality of life, seizure

## Abstract

Epilepsy is primarily defined by the repetitive occurrence of seizures, but the full impact of seizures extends beyond these episodic events. Seizures themselves cause changes at the cellular, network, and systemic levels in individual patients with epilepsy and may contribute to the progressive nature of the disease in some patients. Seizures may have an impact on brain structure and function over time. There is also a high societal and economic burden associated with persistent seizures, including major impacts on quality of life, activities of daily living, and productivity. Therefore, seizure freedom with no or minimal side effects should be the key goal in the treatment of epilepsy, with the potential to reduce both disease progression and the societal and economic impacts of the disease. Physicians have a responsibility to address the key obstacles to early seizure freedom, including optimizing initial treatment strategies, minimizing treatment delays, overcoming therapeutic nihilism, and improving medication adherence.

**Plain Language Summary:**

Epilepsy involves repeated seizures which can cause changes in the brain over time and affect memory, thoughts, and everyday activities. Repeated seizures place a heavy burden on individuals, families, and society, affecting their quality of life, independence, and work. The main goal of epilepsy treatment should be the elimination of all seizures with few or no side effects. To achieve this, doctors should act quickly, choose the right treatments early, avoid treatment delays, and support patients in taking their medications.

**Key points**

- In patients with epilepsy, repetitive seizures have effects at the cellular, network, and systemic levels.
- Repetitive seizures can alter brain structure and potentially contribute to disease progression.
- Seizure freedom with minimal side effects should therefore be the primary goal of epilepsy treatment.
- Achieving seizure freedom offers patients the chance to avoid negative impacts on QoL, independence, and work.
- To target seizure freedom, physicians should aim to optimize treatment, minimize delays, and improve medication adherence.

## 1 INTRODUCTION

Epilepsy is a chronic neurological disorder characterized by recurrent seizures. John Hughlings Jackson historically described seizures as explosive disruptions of brain activity, now recognized as abnormal electrical discharges that disrupt normal neural function. Early in the characterization of seizures, William R. Gowers proposed the ‘seizures‐beget‐seizures’ theory, whereby seizure activity itself is a trigger for changes in the brain that promote the generation of further seizures.^1^ Although this concept has been debated since, there has been growing support for this theory from animal models of epilepsy and from observations in the human brain.^2^, ^3^, ^4^, ^5^, ^6^ In animal models, prolonged, repetitive stimulation of the brain leads to acute changes in brain excitability, leading to the development of self‐sustaining status epilepticus.^2^, ^3^ While status epilepticus is probably the most extreme and sustained manifestation of epileptic activity, even brief episodes of excessive neural activity can affect brain excitability if they are repeated, as demonstrated by the “kindling” phenomenon whereby subthreshold stimulation of the brain over time leads to increased network excitability, induced seizures, and eventually spontaneous seizures (epilepsy).^4^

A hallmark of seizures is that they often occur in clusters interspaced by longer periods of seizure‐free activity. In this regard, the type of seizure may be an important determinant of seizure frequency. In a tetanus toxin model of temporal lobe epilepsy, repeated non‐convulsive seizures appear to trigger seizure clusters, consistent with the seizures‐beget‐seizures model.^5^

The mechanisms by which seizure activity affects brain function are still being investigated, but it is becoming clearer that seizures beget more than seizures. A growing body of evidence suggests that seizures serve as a trigger for a series of cellular and network alterations, including changes in receptor expression, oxidative stress, neuroinflammation, and hyperexcitability that can lead to chronic changes in brain structure and function. These changes may not only increase the likelihood of further seizures but may also lead to structural changes and contribute to cognitive and psychiatric comorbidities that increase the burden of epilepsy on individual patients, caregivers, and society.^1^, ^7^, ^8^, ^9^, ^10^ Here, we summarize the impact of seizures at the cellular/network level, on human brain structure and function, and at the individual–societal level.

## 2 IMPACT OF SEIZURES AT THE CELLULAR/NETWORK LEVEL

There is overwhelming evidence that prolonged seizure activity results in the generation of reactive oxygen species, neuroinflammation, breakdown of the blood–brain barrier, and acute changes to cellular processes, synaptic receptors, ion channels, and neural circuits within the brain (as detailed below). The extent to which briefer seizures or recurrent seizure activity contribute to these effects is still a matter of debate and may be dependent upon factors such as seizure type, epilepsy etiology, and age.

### 2.1 Reactive oxygen species

In vitro studies have demonstrated that prolonged seizure‐like activity is accompanied by N‐methyl‐D‐aspartate (NMDA) receptor activation and consequent large, transient elevations in intracellular free calcium ions.^11^, ^12^ This rise in calcium activates a number of enzymes, including nitric oxide synthase, calpains, and NADPH oxidase, leading to the accumulation of reactive oxygen species (ROS) and potentially neuronal cell death.^13^ NMDA receptor activation is also able to activate NADPH oxidase in a calcium‐independent manner, leading to a rapid and linear accumulation of intracellular ROS and exacerbating metabolic strain on neurons.^12^ The buildup of ROS leads to morphological changes in mitochondria, mitochondrial dysfunction, cytochrome c release, caspase activation, and ultimately programmed necrosis.^14^, ^15^, ^16^ Such neuronal loss is evident in the hippocampus in animal models of status epilepticus.^17^ The involvement of ROS in epilepsy has sparked interest in potential new targets for anti‐epileptogenic therapies, including the transcription factor ‘nuclear factor (erythroid‐derived 2)‐like 2’ (Nrf2), which regulates the production of many antioxidant enzymes.^18^ In animal models, Nrf2 pathway activation has shown antiseizure activity and positive effects on seizure‐related cognitive impairment,^19^ and Nrf2 overexpression has been shown to reduce the number and duration of generalized seizures.^20^

### 2.2 Neuroinflammation

**FIGURE 1:**
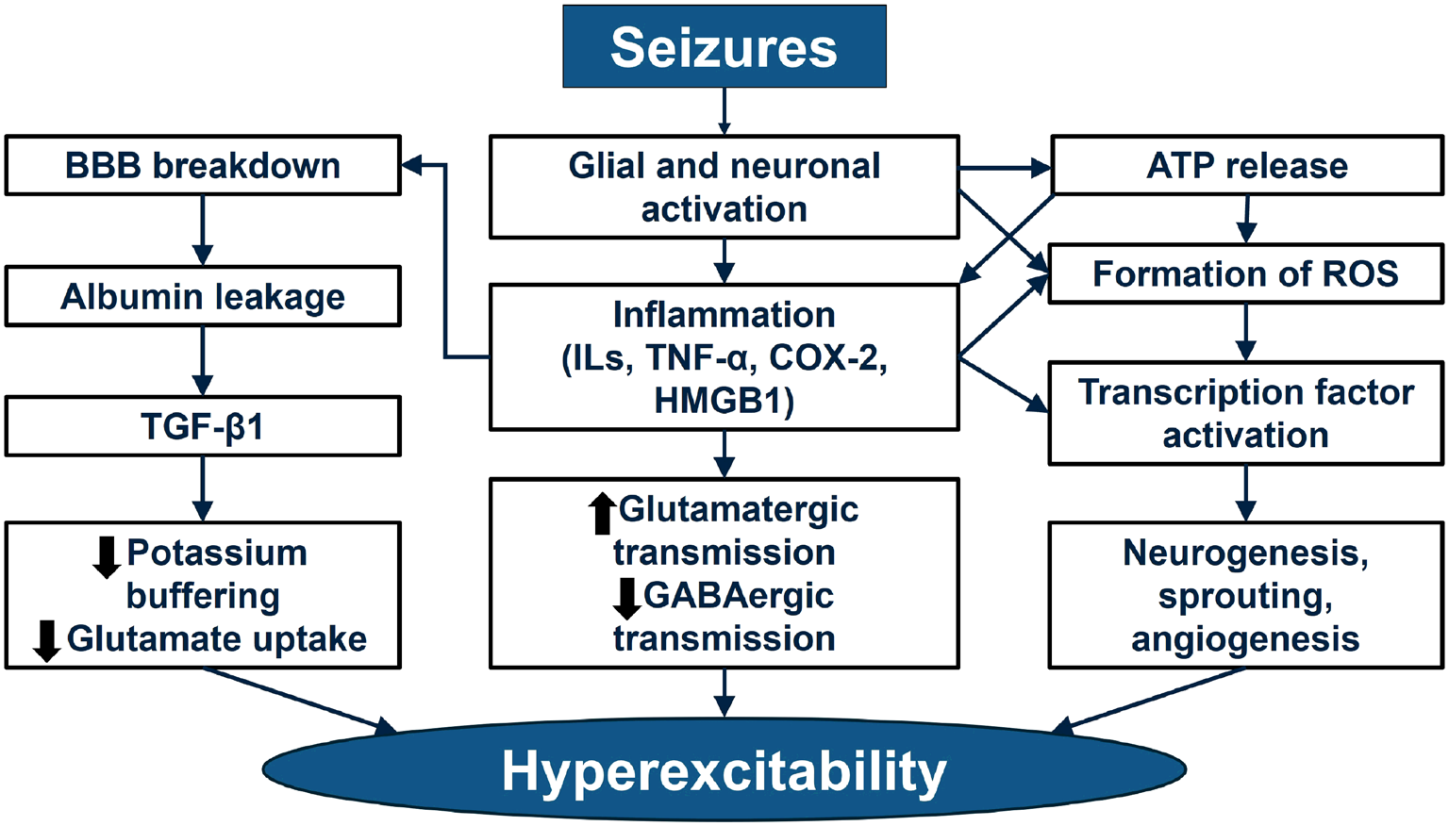
Impact of seizures on hyperexcitability. Seizures trigger the activation of glial cells and neurons, leading to ATP release and the generation of ROS. This process is accompanied by inflammation, which is closely interconnected with other pathological changes, including early breakdown of the blood–brain barrier, leakage of albumin, activation of TGF‐β1, impaired potassium buffering, and reduced glutamate uptake. Inflammation further exacerbates these effects by increasing glutamatergic transmission, reducing GABAergic transmission, and activating transcription factors. This activation drives neurogenesis, sprouting, and angiogenesis, and contributes to hyperexcitability. BBB, blood–brain barrier; COX‐2, cyclooxygenase‐2; GABA, γ‐aminobutyric acid; HMGB1, high mobility group box 1; ILs, interleukins; ROS, reactive oxygen species; TGF‐β1, transforming growth factor‐β1; TNF‐α, tumor necrosis factor α.

Closely associated with the generation of ROS, a substantial body of evidence indicates that seizure activity has a proinflammatory effect in epilepsy.^21^ In rodent models of status epilepticus, seizures trigger activation of glial cells (including astrocytes and microglia) and neurons, leading to expression of proinflammatory cytokines (e.g., IL‐1β, IL‐6, and TNF‐α) and upregulation of cytokine receptors.^22^ This activation triggers a cascade of events, including ATP release, the generation of ROS, and pathological changes, including early breakdown of the blood–brain barrier, leakage of albumin, activation of TGF‐β1, impaired potassium buffering, and reduced glutamate uptake. In addition, inflammation increases glutamatergic transmission, reduces GABAergic transmission, and activates transcription factors leading to neurogenesis, sprouting, and angiogenesis.^13^, ^21^ Together, these effects contribute to the hyperexcitability of neural networks (Figure 1). There is also evidence of an interplay between seizures and peripheral inflammation. For example, disruption of the blood–brain barrier has been shown to stimulate seizure activity in response to peripheral inflammation in a guinea pig whole‐brain model.^23^

Direct evidence of seizure‐induced inflammation in the human brain is limited, but inflammation at the site of the epileptic focus has been reported in a patient with focal epilepsy using positron emission tomography to detect activated microglia. This study observed even greater inflammation at 36 hours following seizure activity, suggesting both a transient and chronic effect of seizures on inflammation.^24^ Moreover, along with inflammation comes the breakdown of the blood–brain barrier, leakage of albumin into the brain, and activation of transforming growth factor beta 1. This directly affects potassium buffering and glutamate uptake and can further contribute to hyperexcitability.^25^ In addition, elevated levels of monocytes, macrophages, and other cells of the innate immune response have been detected in hippocampal tissue from patients with temporal lobe epilepsy and in the hippocampus of patients who have died after status epilepticus.^26^ Higher levels of peripheral inflammation, as shown by increased serum inflammatory biomarkers and enhanced release of inflammatory cytokines from circulating monocytes, have been reported in patients with mesial temporal lobe epilepsy compared with healthy controls.^27^

### 2.3 Connectivity and network effects

Seizures also induce connectivity changes in the brain. A prominent effect is the sprouting of mossy fibers in the hippocampus following prolonged seizure activity, which creates abnormal excitatory circuits and contributes to network hyperexcitability.^28^, ^29^ Changes in the expression and kinetics of synaptic receptors have also been recorded, both in epilepsy models and in neocortical tissue from patients with focal epilepsy. These include increases in α‐amino‐3‐hydroxy‐5‐methyl‐4‐isoxazolepropionic acid (AMPA) receptor expression, changes in the kinetics of NMDA receptor currents, and the appearance of kainate receptors with prolonged postsynaptic currents, each of which leads to heightened excitation.^30^, ^31^, ^32^

We are also beginning to understand the impact of different seizure types at the macro level based on imaging and electroencephalographic recordings. For example, brain network hubs characterized by dense connections to other brain regions have been shown to undergo reorganization in patients with mesial temporal lobe epilepsy, resulting in disruption of distant connections and the emergence of local connections.^33^ Preliminary evidence from patients with status epilepticus indicates the existence of a distinct network that correlates spatially with gene distributions for HOMER2 and metabotropic glutamate receptors 5 and 7.^34^ These data suggest that network mapping may be useful in identifying new targets.

### 2.4 Altered inhibitory mechanisms

Epilepsy is also associated with altered inhibitory mechanisms within the brain. In this respect, the potassium‐chloride cotransporter‐2 (KCC2) plays a pivotal role in regulating inhibitory pathways by extruding chloride and potassium ions following GABA(A) receptor activation. Reductions in expression or function of KCC2 in neurons can result in a buildup of intracellular chloride ions and depolarizing (excitatory) GABA(A) receptor potential.^35^ Paradoxically, overexpression of KCC2 may also have an excitatory effect on neurons by contributing to excessive extracellular potassium buildup.^36^

Neuronal inhibition may also be altered by changes in interneuronal populations. In a model of temporal lobe epilepsy, GABAergic inhibition was found to be reduced in the dendrites of pyramidal neurons due to the loss of dendritic‐projecting interneurons. However, inhibition was increased in the soma resulting from hyperactivity of interneurons projecting to this region.^37^ These effects could potentially reduce the threshold for seizures while promoting their synchronization.

### 2.5 Altered neuronal behavior

In addition to synaptic and structural changes to neurons, seizure activity has been shown to alter neuronal behavior due to changes in the expression of specific ion channels.^38^, ^39^, ^40^ For example, overexpression of T‐type calcium channels following a single episode of status epilepticus has been shown to lead to burst firing of pyramidal neurons in a pilocarpine model.^38^ In a model of temporal lobe epilepsy, neuronal excitability was increased following seizure activity due to a reduction in A‐type potassium channels.^39^ Similarly, loss of HCN channels following a single seizure in a rat model of chronic epilepsy has been associated with enhanced spontaneous activity of pyramidal neurons in the entorhinal cortex.^40^

Seizure‐induced cellular, synaptic, and structural changes are believed to create a positive feedback loop that increases neuronal excitability and the likelihood of further seizures. The combination of these alterations in excitatory and inhibitory networks is likely to contribute significantly to the progression of epilepsy and its comorbidities.

## 3 IMPACT OF SEIZURES ON HUMAN BRAIN STRUCTURE AND FUNCTION

Neuroimaging studies have revealed that epilepsy is associated with widespread brain shrinkage, especially in regions closely interconnected with the epileptic focus. Approximately 75% of patients with focal epilepsy under follow‐up in a large tertiary hospital had accelerated cortical thinning, which affected large areas of the cortex across both hemispheres.^41^ While this process is a feature of disease‐free aging, patients with focal epilepsy show at least double the rate of cortical thinning compared with healthy age‐matched volunteers.^41^ Those patients with epilepsy who have cognitive disturbances show distinct patterns of cortical atrophy, suggesting that cortical thinning is associated with cognitive impairment.^41^, ^42^

In patients with temporal lobe epilepsy, the sequence of atrophy appears to start in the hippocampus and progress, via the thalamus, to the cortex.^43^, ^44^, ^45^ Areas of the cortex most affected by atrophy appear to be those with connectivity to the hippocampus, emphasizing the central role of the hippocampus at the heart of temporal lobe epilepsy.^41^, ^46^ Studies focused on siblings with and without temporal lobe epilepsy suggest that mild hippocampal abnormalities may be shared between siblings, perhaps suggesting an underlying genetic component, but cortical abnormalities appear to be acquired only by those siblings with epilepsy.^47^, ^48^

Different types and localizations of epilepsies are associated with distinct trajectories of brain shrinkage. Xiao et al. described three subtypes of epilepsy based on the pattern of brain atrophy, including two subtypes common to focal and idiopathic generalized epilepsies in which gray matter atrophy was driven by the cortex or the basal ganglia, and a third subtype observed in focal epilepsies characterized by hippocampal atrophy.^44^

Brain atrophy is particularly severe and accelerated during and shortly after status epilepticus. Patients with super‐refractory status epilepticus had a 23% increase in ventricular‐brain ratio, suggesting rapid and marked brain atrophy.^49^ The rate of change was related to the duration of anesthetic agent use,^49^ implying that those with a more severe disease requiring prolonged treatments are at the highest risk of brain atrophy. Another study observed a 16% reduction in the ventricular‐brain ratio in those with status epilepticus and peri‐ictal MRI abnormalities.^50^ The rate of progression of these brain changes underscores the need for early and aggressive intervention to halt or slow the progression of the disease.

This process seems to be preventable by successful treatment of epilepsy. In patients with temporal lobe epilepsy, resective surgery of epileptic foci has been shown to prevent progressive cortical thinning, suggesting that interruption of the underlying epileptic network is critical in preventing further brain damage.^51^ Studies are currently underway to investigate whether antiseizure medications (ASMs) provide a similar protective effect on cortical thinning using brain MRI as a potential biomarker.

## 4 IMPACT OF SEIZURES AT THE INDIVIDUAL–SOCIETAL LEVEL

**FIGURE 2:**
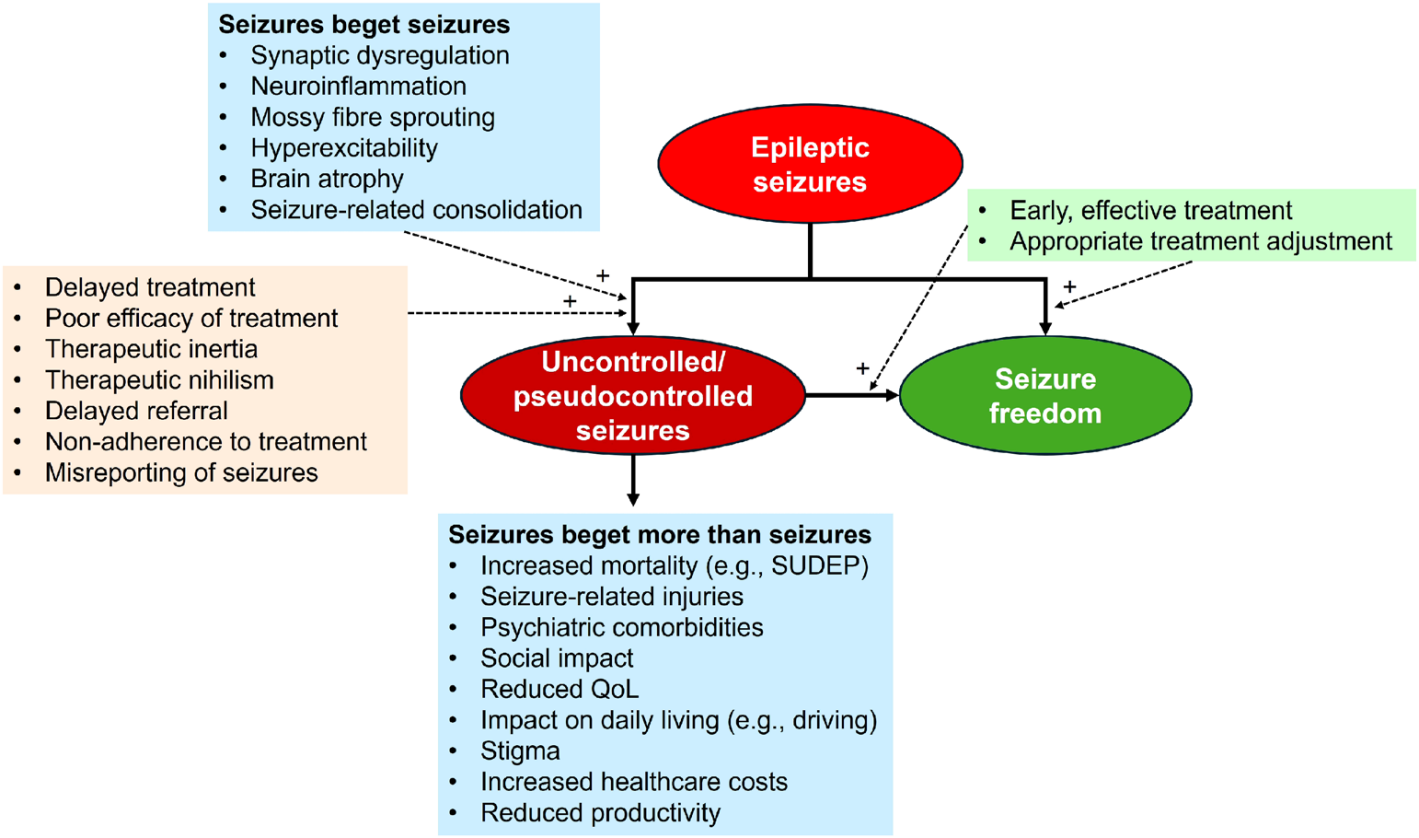
Seizures beget more than seizures: Propagation of seizure activity and downstream consequences. QoL, quality of life; SUDEP, sudden unexpected death in epilepsy.

Epilepsy imposes a significant burden both on individuals and society, particularly due to the high proportion of patients with uncontrolled epilepsy and those labeled as “controlled” but who continue to have seizures.^52^ In 2021, active idiopathic epilepsy led to almost 14 million disability‐adjusted life‐years, prompting the World Health Organization to declare epilepsy a top‐priority non‐communicable disease in 2022.^53^ In addition to their effect on seizure recurrence, persistent seizures have multiple impacts on patients and the healthcare system, including mortality, seizure‐related injuries, comorbidities, reduced quality of life (QoL), impaired activities of daily living, and increased costs (Figure 2).^54^, ^55^, ^56^, ^57^, ^58^, ^59^

### 4.1 Mortality

Patients with epilepsy typically live 10–12 years less than the general population,^60^ and the risk of mortality is 40‐fold higher in patients with uncontrolled seizures compared with those in remission.^61^ Sudden unexpected death in epilepsy (SUDEP), status epilepticus, and drowning are the main causes of increased mortality in these patients.^62^

SUDEP, the most common cause of epilepsy‐related death in children and adults, occurs in approximately 1 per 1000 patients with epilepsy each year,^63^ and in up to 9.3 per 1000 patients in epilepsy‐surgery candidates with drug‐refractory epilepsies.^61^ While SUDEP is considered relatively rare, it is probably under‐reported. A US study found that SUDEP accounted for approximately 5.3% of sudden deaths in individuals aged 18–64, although only 1.5% were attributed to seizures or complications of seizures on death certificates.^64^ SUDEP is also a substantial public health burden as it often occurs in young patients, leading to a high number of life‐years lost.^65^

Several studies have investigated risk factors for SUDEP.^66^, ^67^, ^68^ The occurrence of generalized tonic–clonic (GTC) seizures is the most significant risk factor, leading to a 10‐fold increased risk of SUDEP, rising to a 15.5‐fold increased risk in those with three or more GTC seizures per year.^67^ Various SUDEP risk‐assessment scales, such as SUDEP‐3, SUDEP‐7, and SUDEP‐CARE, have been developed to predict SUDEP.^69^, ^70^ Among them, SUDEP‐3, which evaluates risk based on the frequency of GTC seizures, overall seizure frequency, and the presence of intellectual disability, appears to be more effective than SUDEP‐7 in predicting SUDEP outcomes.^70^, ^71^

Notably, the incidence of SUDEP is associated with increasing refractoriness of disease, suggesting that interventions that successfully reduce seizures may have a role in reducing the incidence of SUDEP.^54^ Indeed, a meta‐analysis of 112 placebo‐controlled studies found that patients with drug‐resistant epilepsy who received adjunctive therapy with active ASMs had a 7‐fold lower risk of SUDEP compared with those receiving a placebo.^68^

Status epilepticus accounts for approximately 10% of deaths associated with epilepsy, with fatality rates varying between 5% and 39% across studies.^72^ Factors associated with mortality during status epilepticus include the cause of status epilepticus, its duration, and the age of the patient.^72^, ^73^

The incidence of drowning in individuals with epilepsy is 10–20 times higher than in the general population,^74^, ^75^ with most deaths occurring in bathtubs.^75^

### 4.2 Fractures and other seizure‐related injuries

A frequently overlooked issue for patients with epilepsy is the occurrence of seizure‐related injuries. Bone fractures associated with generalized convulsive seizures and status epilepticus typically occur in the skull, shoulders, and vertebrae.^76^ The prevalence of fractures among patients with epilepsy is high (approximately 1 in 10 patients over a 3‐year period), and those with specific comorbidities (e.g., depression, stroke, and dementia) are at the highest risk.^77^ Notably, fractures that appear after seizures are often the result of tonic muscular contraction rather than a specific trauma or fall,^78^ emphasizing the need to take account of changes in pain and daily function following seizures.

The risk of seizure‐related injuries is directly related to both seizure type (especially GTC seizures) and seizure frequency.^79^ Individuals with untreated or inadequately treated epilepsy have an 8–16‐fold higher risk of epilepsy‐related injuries and typically experience more severe outcomes compared with individuals receiving evidence‐based treatment.^55^ Therefore, while some level of injury risk is always present, appropriate evidence‐based treatment can substantially reduce this risk, improving overall safety and QoL for individuals with epilepsy.

It should be noted, however, that some ASMs can have a significant negative impact on bone health. Carbamazepine, for example, is associated with reduced levels of vitamin D and calcium, and increased levels of alkaline phosphatase in patients with epilepsy, suggesting that long‐term treatment with carbamazepine may be deleterious in patients with osteoporosis or other skeletal diseases.^80^

### 4.3 Psychiatric comorbidities, mood, and stigma

The impact of seizures on psychiatric comorbidities is well documented. A Canadian study reported that 32% of patients with epilepsy who had experienced at least one seizure within the previous 5 years had a 4‐fold higher incidence of major depressive symptoms than patients who were seizure‐free over this period.^56^ These patients had an increased risk of mood disorders and experienced a greater degree of stigma compared with seizure‐free patients.^56^ In a Spanish study, depressive symptoms were reported in 62–65% of patients with refractory focal epilepsy compared with 32–37% of patients with controlled disease.^57^ A similar study of patients with focal epilepsy found that those who achieved seizure freedom showed a significantly lower incidence of major depressive symptoms (8 vs. 23%) than patients with persistent seizures.^58^ In addition, patients with uncontrolled seizures experience psychological and social dysfunction, employment difficulties, and reduced QoL.^8^, ^81^, ^82^, ^83^ These impacts may be related to the cognitive decline that is prevalent in patients with epilepsy, alongside feelings of anxiety associated with the expectation of further seizures.^84^

### 4.4 Quality of life

The impact of uncontrolled epilepsy on patient QoL is substantial and is compounded by psychiatric comorbidities, which themselves impact QoL.^85^ In Europe, patients with epilepsy show significantly lower QoL scores across multiple physical and mental domains compared with the normal population.^86^

Risk factors associated with reduced QoL include earlier age at epilepsy onset, a higher frequency of seizures, and longer seizure duration,^8^, ^87^, ^88^ whereas the achievement and maintenance of seizure freedom have been shown to be key factors for improvement in QoL.^58^, ^89^ Moreover, even patients with a 75–99% reduction in seizure frequency report worse QoL scores than patients who are seizure‐free.^59^

Adverse effects associated with treatment are also a significant determinant of QoL in patients with drug‐resistant epilepsy.^85^, ^90^ Notably, these effects vary between ASMs, as different medications can affect comorbidities that impact QoL in distinct ways.^91^, ^92^

### 4.5 Impact on daily living

Uncontrolled epilepsy also has a significant impact on patients' normal activities of daily living. Patients with seizures experience greater socioeconomic barriers and limitations in daily activities compared with seizure‐free patients.^56^ In this regard, patients report restrictions on driving eligibility as the most challenging impact, alongside reduced employment opportunities, dangers associated with seizures, and adverse effects of ASMs.^93^ Therefore, when choosing treatments for their condition, patients rate seizure freedom and the preservation of cognitive function as the most important influencing factors.^94^

For patients with newly diagnosed epilepsy who are waiting to achieve seizure freedom, several challenges have been identified that impact daily living, including mental health struggles, a feeling that their lives are on hold, difficulty navigating the healthcare system, and challenges in finding the appropriate healthcare professional to meet their needs.^95^

A systematic review has shown that seizure emergencies have a significant negative impact on short‐term QoL and activities of daily living, citing physical injury and financial burden to the patient as the most prominent features.^96^ Notably, acute treatments were found to reduce the short‐term burden of seizures, leading to a rapid return to normal function for patients and caregivers.^96^

### 4.6 Costs

From an economic and societal perspective, the costs of epilepsy are substantial. Most hospital admissions (95%) are for acute disease, and costs for hospitalizations are high.^83^ Furthermore, a German study found that direct costs, including hospitalizations and ASMs, accounted for only a third of the overall financial burden, whereas indirect costs, primarily due to lost productivity and the inability of many patients to maintain full‐time employment, accounted for the remaining two‐thirds.^82^

Both direct and indirect costs are significantly affected according to whether patients achieve freedom from seizures. In Germany, direct costs are 2‐fold higher for patients with active epilepsy compared with seizure‐free patients, including a 5‐fold higher cost of hospitalization.^97^ Similarly, in the USA, annual direct costs are reportedly over 75% higher for patients with refractory versus non‐refractory epilepsy.^98^ In Austria, lack of seizure freedom has been shown to impart an approximately 2‐fold higher total cost of epilepsy, driven largely by unemployment,^99^ and patients with active epilepsy in Germany incur a 2‐fold higher indirect cost associated with days off work compared with seizure‐free patients.^97^

Despite the introduction of new ASMs, the distribution between direct (including the cost of ASMs) and indirect costs of active epilepsy has remained relatively stable over the last 20 years.^82^ In Germany, while the cost of newer, more effective ASMs has risen, the overall expense (i.e., budget impact) associated with epilepsy treatment has declined due to the widespread use of generics such as lamotrigine and levetiracetam.^100^ Indeed, following the US launch of cenobamate and fenfluramine for focal epilepsy and Dravet syndrome, respectively, fewer than 5% of eligible patients were treated with these new ASMs in the 2 years after market entry, despite their greater efficacy and good tolerability profile compared with generic ASMs.^101^ This may represent a lost opportunity, considering the impact of uncontrolled seizures on brain function and the relatively low budget impact of new ASMs on overall costs.

## 5 BARRIERS TO ACHIEVING SEIZURE FREEDOM

As described above, failure to achieve seizure freedom in patients with epilepsy impacts mortality, seizure‐related injuries, comorbidities, QoL, activities of daily living, and costs.^54^, ^55^, ^56^, ^57^, ^58^, ^59^ However, approximately 30% of individuals with epilepsy do not achieve seizure freedom, despite treatment with multiple ASMs.^98^ The reasons for this failure are multifactorial.

Among healthcare providers (HCPs), therapeutic inertia can occur, in which there is a failure to optimize treatment due to misperceptions of epilepsy control (either among physicians or their patients), reluctance to change ASM regimens, or doubts regarding efficacy.^102^ This can lead to therapeutic nihilism, in which treatment is withheld due to perceived futility.^103^ Both cases may result in persistent seizures, with the downstream impacts described above. A significant factor in this regard is that early epilepsy treatment is often provided by general neurologists who are not specialized in epilepsy and may not be familiar with the latest, most effective treatment options. Although patients who achieve early seizure freedom have a more favorable prognosis than those with persistent seizures,^56^, ^59^ delays in optimizing and switching ASM treatment occur.^104^, ^105^ Additionally, referrals to specialist care can be delayed,^106^ despite evidence showing that timely referral reduces seizure frequency and improves seizure‐freedom rates.^107^

Among patients, non‐adherence to medication contributes to suboptimal treatment of epilepsy.^108^, ^109^, ^110^ Non‐adherence rates of 26–79% have been reported in studies of patients with uncontrolled epilepsy,^108^, ^109^, ^110^ and higher rates have been reported with old versus new ASMs.^110^ In patients with uncontrolled epilepsy, non‐adherence can lead to increased seizure recurrence, status epilepticus, a greater incidence of injuries and fatalities, and more hospitalizations.^109^ Moreover, up to 45% of seizures may be unreported by patients,^111^ potentially leading to undertreatment. Addressing these barriers is crucial for optimizing seizure‐freedom rates.

In addition, several intrinsic biological factors may reduce the likelihood of achieving seizure freedom, including alteration of drug targets or drug uptake in the brain, abnormal neural networks, genetic factors (e.g., *SCN1A* mutations), epigenetic variation, neuroinflammation, blood–brain barrier dysfunction, and psychiatric comorbidities.^112^

## 6 FUTURE CONSIDERATIONS

The numerous downstream processes triggered by recurrent seizures that contribute to disease progression, neurological impairment, and worsening comorbidities underscore the need for early and effective treatment to eliminate seizures. In this regard, it will be important to develop an understanding of how different treatment options impact these processes in order to improve efficacy and reduce comorbidities. In patients with newly diagnosed epilepsy, preliminary studies using functional MRI have observed early brain network changes driven by recurrent seizures, which appear to be reduced in those patients responding to ASMs.^113^ Developing biomarkers to monitor disease activity and progression is a key goal in epilepsy research, as current regulatory clinical trials rely heavily on unreliable seizure counts as measures of efficacy. Such biomarkers would support more precise identification of the most effective ASMs for individual cases and could form the foundation of personalized epilepsy treatment.

An understanding of downstream events following seizures may also provide an opportunity to identify novel targets for intervention. For example, mapping of neuroinflammatory pathways associated with seizures has provided a number of potential molecular and cellular targets for new treatments, alongside potential biomarkers for epilepsy research.^114^ Similarly, microRNAs are now being considered as potential tools for regulating neurotransmitter receptor expression and hyperexcitability.^115^ Seizure‐related consolidation (SRC) may offer another opportunity for anti‐epileptogenic intervention. SRC is a process by which neural pathways activated at the start of seizures are reactivated during post‐seizure sleep, leading to persistent changes in neural activity.^116^ Therapies that target slow‐wave and rapid‐eye‐movement sleep following seizures could potentially reduce the future frequency and severity of seizures.^116^

## 7 CONCLUSION

Seizures are not simply episodic events but should be considered as catalysts for a cascade of cellular, structural, and systemic changes in the brain that may contribute to the progressive nature of epilepsy. These changes can increase neuronal excitability, promote brain atrophy, and exacerbate cognitive and psychiatric comorbidities. In addition, the societal and economic burden of ongoing seizures remains high, with significant costs associated with treatment and lost productivity. Since ongoing seizures are the driving force of these processes and downstream effects, physicians should prioritize achieving early and complete seizure freedom for their patients. This should not only slow the potential progression of disease, but also reduce the extensive impact of seizures on individuals and society. To support this goal, it is essential to address the modifiable barriers to early seizure freedom, such as increasing awareness of the most effective available treatments within primary care, reducing treatment delays, overcoming therapeutic nihilism, and improving adherence to medication.

## AUTHOR CONTRIBUTIONS

All authors contributed equally to this study, including review of the literature, analysis and interpretation of data, drafting and/or critically revising the article with respect to intellectual content. All authors have read and approved the final manuscript version to be published.

## FUNDING INFORMATION

No funding was received for this research.

## CONFLICT OF INTEREST STATEMENT

Matthew Walker has acted as a consultant for Seer and EpilepsyGtx, is a founder shareholder of EpilepsyGtx, and has received honoraria from Angelini Pharma, Eisai, and UCB Pharma. Marian Galovic has received honoraria for advisory and/or educational activities from Angelini Pharma, Arvelle, Eisai, Nestlé Health Science, Neuraxpharm, and UCB Pharma. Elena Álvarez‐Barón is an employee of Angelini Pharma. Adam Strzelczyk has received personal fees and grants from Angelini Pharma, Biocodex, Desitin Arzneimittel, Eisai, Jazz Pharmaceuticals, Longboard, Neuraxpharm, Stoke Therapeutics, Takeda, UCB Pharma, and UNEEG Medical.

## ETHICS STATEMENT

We confirm that we have read the Journal's position on issues involved in ethical publication and affirm that this report is consistent with those guidelines.

## Data Availability

The data that support the findings of this study are available from the corresponding author upon reasonable request.
